# Two-dimensional SiC/AlN based type-II van der Waals heterobilayer as a promising photocatalyst for overall water disassociation

**DOI:** 10.1038/s41598-022-24663-y

**Published:** 2022-11-22

**Authors:** Naim Ferdous, Md. Sherajul Islam, Jeshurun Biney, Catherine Stampfl, Jeongwon Park

**Affiliations:** 1 Department of Electrical and Biomedical Engineering, University of Nevada, Reno, NV 89557 USA; 2 Department of Electrical and Electronic Engineering, Khulna University of Engineering and Technology, Khulna, 9203 Bangladesh; 3 School of Physics, The University of Sydney, Sydney, NSW 2006 Australia; 4 School of Electrical Engineering and Computer Science, University of Ottawa, Ottawa, ON K1N6N5 Canada

**Keywords:** Energy science and technology, Materials science, Nanoscience and technology, Nanoscale devices, Nanoscale materials

## Abstract

Two-dimensional (2D) van der Waals (vdW) heterostructures made by vertical assembling of two different layers have drawn immense attention in the photocatalytic water disassociation process. Herein, we suggest a novel 2D/2D vdW heterobilayer consisting of silicon carbide (SiC) and aluminum nitride (AlN) as an exciting photocatalyst for solar-to-hydrogen conversion reactions using first-principles calculations. Notably, the heterostructure presents an inherent type-II band orientation wherein the photogenic holes and electrons are spatially separated in the SiC layer and the AlN layer, respectively. Our results indicate that the SiC/AlN heterostructure occupies a suitable band-gap of 2.97 eV which straddles the kinetic overpotentials of the hydrogen production reaction and oxygen production reaction. Importantly, the built-in electric field at the interface created by substantial charge transfer prohibits carrier recombination and further improves the photocatalytic performance. The heterostructure has an ample absorption profile ranging from the ultraviolet to the near-infrared regime, while the intensity of the absorption reaches up to 2.16 × 10^5^ cm^−1^. In addition, external strain modulates the optical absorption of the heterostructure effectively. This work provides an intriguing insight into the important features of the SiC/AlN heterostructure and renders useful information on the experimental design of a novel vdW heterostructure for solar energy-driven water disassociation with superior efficiency.

## Introduction

The generation of oxygen and hydrogen by solar energy induced photocatalytic water splitting is one of the most favorable methods for meeting future energy demands^1–3^. However, to develop a high performance water-splitting photocatalysis system, it requires a material with an appropriate band-gap, outstanding carrier separation for effective surface activity, and appropriate redox potentials to drive the redox reaction smoothly, as well as photochemical stability, environmental friendliness, and commercial feasibility^3,4^. Owing to these requirements for the semiconducting materials to be effective photocatalysts and their significance in the water disassociation process, a substantial amount of research has been carried out in this field. Fujishima and Honda were the first to report solar energy driven water disassociation over TiO_2_ in 1972^5^. Following their groundbreaking work, a great many efforts have been made towards investigating efficient photocatalytic materials and significant numbers of photocatalysts including conjugated polymers^6–8^, oxyhalides^9^, oxynitrides^10^, metal chalcogenides, and metal oxides^11–13^, and so on are reported. Nevertheless, the practical applicability of these materials is severely limited by their higher electron–hole recombination rate accompanied by the poor absorption profile in the visible regime^14^. In this context, two-dimensional (2D) materials have recently been in the limelight for their capability in photocatalytic water splitting which includes layered transition metal dichalcogenides (TMDs)^15^, Janus TMDs^16,17^, MXene^18^, graphene-like honeycomb BC_3_ single layer^19^, C_3_N_4_^20^, phosphorene^21^, etc. Despite having promising physical and chemical properties together with an extremely high ratio of the surface to the volume, monolayer 2D materials exhibit low quantum efficiency in photocatalysis because of their smaller carrier lifetime due to the photogenic carriers remaining in one region for limited time intervals.

In this regard, type-II van der Waals heterostructures (vdWHs) made by stacking of two different 2D materials have been proposed as a promising solution to this problem^22–24^. As 2D/2D type-II heterobilayers provide spatial separation of the photoexcited electrons and holes in two distinct layers effectively to inhibit carrier recombination, the quantum efficiency of photocatalysis is improved significantly. In addition, these heterostructures can mediate their electronic band structures to satisfy the potential requirements of the solar-to-hydrogen conversion reaction^25^. The inherent electric field facilitates the separation of the majority of photogenic carriers in distinct layers of the structure, prolonging the lifetime of the photo-induced carriers. The quantum confinement in the heterostructures further enriches electron–electron correlation and exciton binding energy. Moreover, 2D vdWHs enable photoexcited electrons and holes to make the largest possible contact surface with water while reducing the gap electrons and holes migrate, decreasing electron–hole recombination rate, and improving photocatalytic performance^25–27^. Additionally, the dynamic overpotentials of the oxygen and hydrogen production reactions are increased due to the intrinsic dipole moment of the vdWHs, which further ensures the photocatalytic water disassociation process.

Over the last few years, numerous theoretical and experimental studies have been devoted to enhancing catalytic performance by creating vdWHs through diverse 2D materials, including SiC/MoS_2_^28^, phosphorene/g-C_3_N_4_^29^, InSe/SiH^30^, SiC/MSSe (M = Mo, W)^31^, AlN/Blue-phosphorene^32^, SiC/BS^33^, AlN/MX_2_ (MX_2_ = MoSe_2_, WS_2_, and WSe_2_)^34^, GaAs/SiH^23^, SiC/GaN^35^, SiC/GeC^36^, MoSe/AlN (GaN)^37^, h-BN/C_2_N^38^, CdO/CdS^39^ and so forth. In particular, 2D silicon carbide (SiC) based bilayer heterostructures have been found to be attractive and exciting candidates for advanced photocatalytic water decomposition reaction which is attributable to the excellent optical characteristics, stable planar structure, high thermal capability, outstanding electrical conductivity, and direct bandgap semiconducting properties of the SiC sheet^40–43^. Chemical inertness, excellent strength, and high saturated mobility of the carriers of the SiC layer have outlined its immense potential as a heterogeneous catalyst^41,44,45^. On the other hand, graphene-like single layered aluminum nitride (AlN), a wide-band-gap semiconductor^46^ has sparked a lot of attention among researchers as a new and classic 2D III-nitride material in optical, spintronic, optoelectronic, and substrate applications^47^. 2D AlN exhibits comparable symmetry and lattice parameter to the SiC monolayer with a little mismatch, suggesting the easy realization of the SiC/AlN heterobilayer in experiments. Recent investigations reveal that SiC can be prepared either by sonicating wurtzite SiC^48^ or by substituting the C atom in a graphene structure with a Si atom^43^. Researchers have successfully prepared a thin SiC structure using a catalyst-free carbothermal technique and a post-sonication procedure^49^. A new experiment on true 2D SiC exfoliated successfully from bulk SiC was published recently by Chabi et al*.*^50^. Moreover, it has been found possible to produce high purity monolayer films of AlN using standard growth procedures^51,52^. Nevertheless, heterostructures consisting of layered SiC and AlN sheets have not been analyzed in the literature to assess their potential for photocatalytic activity. Hence, it is worthwhile to adapt the 2D/2D vdWH comprising of the SiC and AlN layer and reveal its photocatalytic water decomposition performance.

In this article, we propose a type-II vdWH consisting of 2D SiC/AlN as an efficient photocatalyst for water disassociation using first-principles calculations. We consider six likely configurations and study their viability in the hydrogen production reaction and the oxygen production reaction by investigating structural, electronic, interfacial, and optical properties. The band edge positions of the SiC/AIN bilayer heterostructure straddle the redox reaction of water decomposition at acidic as well as neutral environment, realizing the overall catalytic reaction. The electric field at the heterostructure interface formed due to significant amount of charge transfer prevents electron–hole recombination, further improving the photocatalytic performance. We also investigated the effect of biaxial strain on the catalytic performance of the SiC/AlN heterobilayer. All these features point toward the superior photocatalytic performance of the SiC/AlN vdWH and provide insights for the experimental design of type-II 2D/2D catalysts based on SiC and AlN sheets.

## Computational details

We conducted first-principles calculations within the framework of density functional theory (DFT) as employed in the *MedeA VASP* (Vienna ab initio simulation package)^53,54^. The projector-augmented-wave (PAW) potentials are used to characterize the core electrons^55^. We considered the optB86b-vdW van der Waals Density Functional (vdW-DF) developed by Klimeš and co-workers^56,57^ for describing the exchange–correlation interaction of electrons owing to its capability of describing the interaction between the SiC and AlN layers effectively. The cutoff energy of the plane-wave basis was taken as 500 eV. We sampled the Brillouin-zone with a $\Gamma$-centered 24 $\times$ 24 $\times$ 1 Monkhorst–Pack (MP) grid^58^ for structural relaxations, electronic and optical properties calculations. A sufficient vacuum region of greater than 15 Å was employed along the Z-axis of the heterostructures in order to avoid the interaction between the two subsequent slabs. Throughout the structural relaxation, the atomic positions and the lattice were relaxed until the total energy (force) converged to 1 $\times$ 10^–5^ eV (0.02 eVÅ^−1^).

The optical properties of the systems can be determined based on the frequency dependent dielectric function $\varepsilon \left(\omega \right)={\varepsilon }_{1}\left(\omega \right)+i{\varepsilon }_{2}\left(\omega \right)$, which characterizes a system’s linear response to the electromagnetic field. Kramers–Kronig relations can be used to determine ${\varepsilon }_{1}$ and ${\varepsilon }_{2}$ ^59^. We can quantify the imaginary part within the long wavelength limit ($q\to 0)$ in independent particle approximation as:

1 $${\epsilon }_{2}\left(\omega \right)={\varepsilon }_{\alpha \beta }^{\left(2\right)}\left(\omega \right)=\frac{4{\pi }^{2}{e}^{2}}{\Omega }\underset{q\to 0}{\mathrm{lim}}\frac{1}{{q}^{2}}\sum_{c,v,k}2{w}_{k}\delta \left({\epsilon }_{ck}-{\epsilon }_{vk}-\omega \right)\times \langle {u}_{ck+{e}_{\alpha }q}|{u}_{vk}\rangle {\langle {u}_{ck+{e}_{\beta }q}|{u}_{vk}\rangle }^{*}$$

Here, the primitive cell’s volume is represented by $\Omega, {w}_{k}$ being the weights of the K point, while the spin degeneracy is described by introducing factor 2 in the summation. The ${\epsilon }_{vk}$ (${\epsilon }_{ck}$) values indicate energies of the k dependent valence (conduction) band, while cell periodic part of the pseudo-wave function are represented by ${u}_{vk}$ and ${u}_{ck}$; ${e}_{\alpha ,\beta }$ being the unit vectors towards the cartesian coordinate directions. We can determine the real part (${\varepsilon }_{1})$ based on the Kramers–Kronig transformation^59^:

2 $${\varepsilon }_{1}\left(\omega \right)={\varepsilon }_{\alpha \beta }^{\left(1\right)}\left(\omega \right)=1+\frac{2}{\pi }P{\int }_{0}^{\infty }\frac{{\varepsilon }_{\alpha \beta }^{\left(2\right)}\left(\omega \right)\left({\omega }^{{{\prime}}}\right)}{{{\omega }^{{{\prime}}}}^{2}{\omega }^{2}+i\eta }d{\omega }^{{{\prime}}}$$

Here, $P$ is the principal value while a trivial complex shifting ($\eta )$ of 0.1 is introduced in order to smoothen the real part (${\varepsilon }_{1})$ a little, which is admissible for most of the computations^60^. The optical spectrum of the material can be calculated using the formula:

3 $$\alpha (\omega )=\frac{\sqrt{2}\omega }{c}{[{\{{\varepsilon }_{1}^{2}(\omega )+{\varepsilon }_{2}^{2}(\omega )\}}^{0.5}-{\varepsilon }_{1}(\omega )]}^{0.5}$$

where $\alpha$ being the absorption coefficient, ${\varepsilon }_{1}^{2}$ and ${\varepsilon }_{2}^{2}$ refer to the dielectric function’s real and the imaginary parts, respectively, $\omega$ stands for angular frequency while *c* is the speed of light in the vacuum.

## Results and discussion

The analysis begins with the electronic characteristics of the constituents, the SiC monolayer, and AlN monolayer. The band structures of the free standing SiC monolayer and the AlN monolayer are illustrated in Fig. S1. The free-standing SiC monolayer reveals semiconducting property with an indirect bandgap of 2.514 eV. The valence band maximum (VBM) is at the high symmetry K point, while the conduction band minimum (CBM) of isolated SiC is located at the M point. The result corresponds well with the study of Hoat et al*.* (indirect band gap of 2.492)^61^ and Peng et al*.*^35^. In addition, the C-2p state dominates in the valence band of the isolated SiC monolayer, while the predominant contribution for the conduction band comes from the Si-3p state^61^. The isolated AlN monolayer also demonstrates semiconducting behavior. In our calculation, we obtained an indirect bandgap of 2.784 eV for single layered AlN. The VBM is positioned at the high symmetry K point whereas the CBM of the pristine AlN layer is at the $\Gamma$ point. This is also in line with the literature^62,63^. Moreover, the VBM of the free-standing AlN layer comes mainly from N p_z_ orbital while the CBM is attributed to N s and Al s orbitals^63^.

The SiC/AlN van der Waals bilayer heterostructure (vdWBH) is achieved through staking the primitive cell of the monolayer AlN above the primitive cell of 2D-SiC. Our calculation yielded the lattice constants of 3.094 Å and 3.126 Å for SiC and AlN, respectively, which are in excellent agreement with the previous theoretical and experimental results^46,47,50,64–71^. Accordingly, a small lattice mismatch (LMM) of ~ 1.02% is present between the SiC and AlN monolayers, as obtained using the formula ${\text{LMM  }} = \left| {\frac{{{\text{a}}_{{{\text{SiC}}}} { - }{\text{a}}_{{{\text{AlN}}}} }}{{{0.5 }\times  ( {{\rm a}}_{{{\text{SiC}}}} +  {\text{ a}}_{{{\text{AlN}}}} {)}}}} \right| \times 100\%$, where a_SiC_ and a_AlN_ are the optimized lattice constants of the 2D-SiC and 2D-AlN, respectively. This small lattice mismatch suggests that the two different layers can be stacked vertically to build the desired heterostructure. As reported by Hu et al*.*^72^, it is promising if the heterostructure is built with a lattice mismatch of less than 5%.

**Figure 1:**
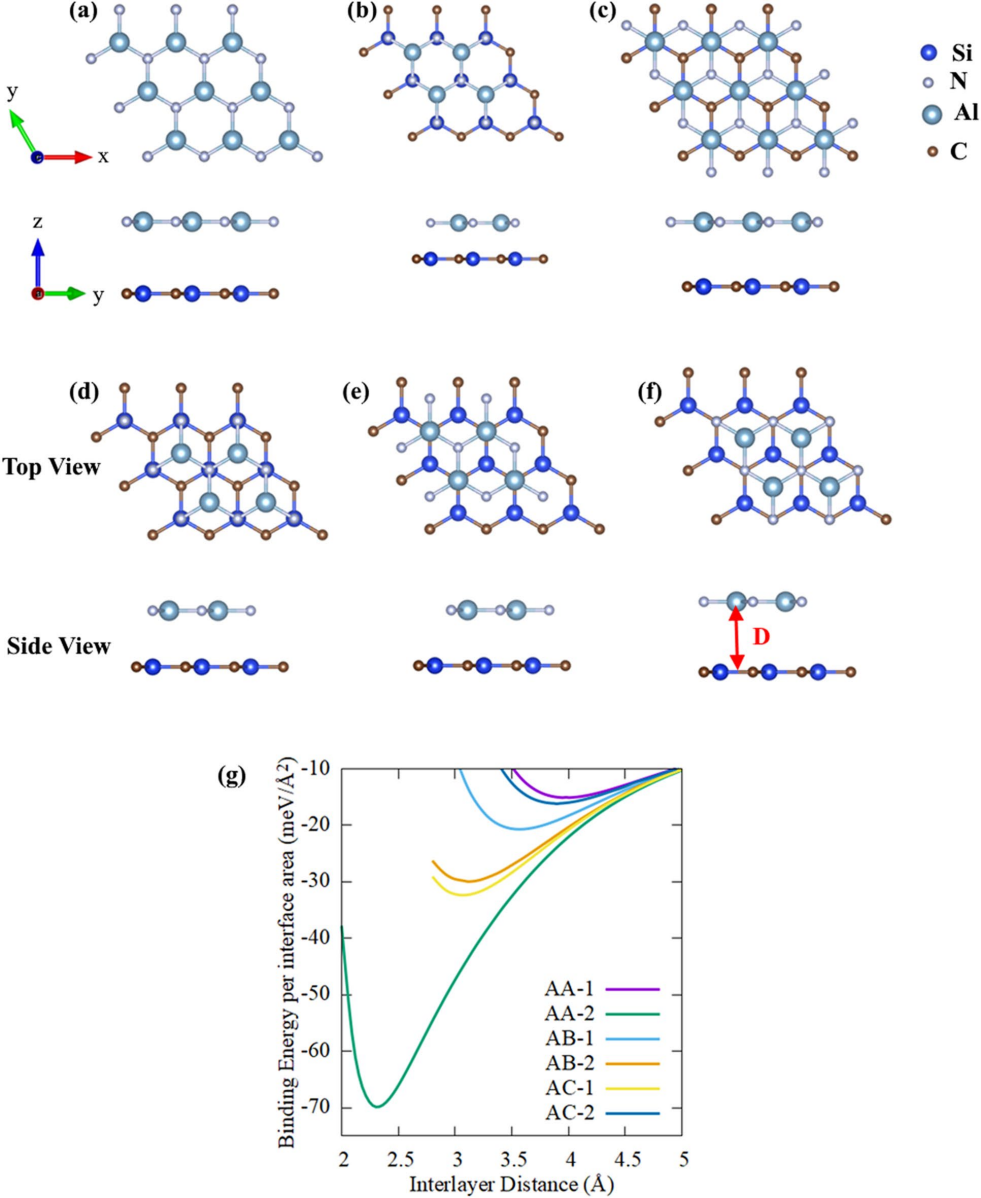
Top and side views of the SiC/AlN bilayer heterostructure in the six configurations considered: (**a**) AA-1, (**b**) AA-2, (**c**) AB-1, (**d**) AB-2, (**e**) AC-1, (**f**) AC-2. ‘D’ represents the equilibrium layer spacing between the SiC layer and the AlN layer. (**g**) Variation of the binding energy per interface area as a function of layer spacing between the SiC and AlN layers for the six stacking configurations.

As structural modification leads to changes in physical properties, we considered six prospective configurations of the SiC/AlN vdWBH to analyze the effect of different stacking geometries. The six optimized stacking configurations are shown in Fig. 1 and marked as AA-1, AA-2, AB-1, AB-2, AC-1 and AC-2. In the AA-1 configuration, the Al and N atom of the AlN layer are placed right above the Si and C atom of the SiC monolayer, respectively while the AA-2 stacking pattern is obtained by pacing the Al and N atom straight above the C and Si atom of the 2D-SiC respectively. Configuration AB-1 (AB-2) is achieved by positioning the Al atom (N atom) vertically above the Si atom of the SiC monolayer whereas the N atom (Al atom) of the 2D-AlN is above the center of the hexagon formed by the SiC layer and C atom is directly below the center of the hexagon formed by the AlN layer. Similarly, we obtained the AC-1 (AC-2) configuration by placing the Al atom (N atom) directly above the C atom of the 2D-SiC whereas the N atom (Al atom) is placed right above the center of the hexagon formed by the SiC monolayer and the Si atom is located straight below the center of the hexagon formed by AlN layer. Our structure of SiC/AlN vdWBH is comparable to the GeC/GaN vdW heterojunction studied by Lou and Lee^26^. Owing to similar lattice parameters of the GeC and GaN layer with a very small lattice mismatch of 0.63%, they formed the heterojunction by stacking the GeC monolayer and GaN monolayer and considered six similar stacked forms to analyze its potential as photo-catalyst for the water-splitting process. Our present vdWBH structure is also analogues to the ZnO/GaN vdW heterostructure ^73^. Owing to a little lattice mismatch of 0.26%, Ren et al*.*^73^ constructed the vertical heterostructure composed of monolayered GaN and ZnO and formed six representative stacking patterns for its theoretical prediction as a photo-catalyst. Besides, while studying 2D GaN/SiC bilayer, Peng et al*.*^35^ also considered the six most likely structures of the GaN/SiC bilayer.

With a view to assessing the relative stability of the SiC/AlN vdWBH efficiently, we estimated the binding energy (BE) of the heterostructure utilizing the expression:

4 $$BE=({E}_{SiC/AlN}-{E}_{SiC}-{E}_{AlN})/A$$

where ${E}_{SiC/AlN}$ stands for the total energy of the SiC/AlN vdWBH, ${E}_{SiC}$ and ${E}_{AlN}$ refer to the energy of the isolated monolayers of SiC and AlN, respectively. ‘$A$’ stands for the interface area. A negative value of binding energy implies that the system is energetically favorable. It also points out toward easy realization of the system. The more negative the binding energy, the more stable the system is. Conversely, a positive binding energy value indicates structural instability. The binding energy values of the six configurations are enlisted in Table S1. As the binding energies of the six configurations suggest, all the stacking patterns are stable while configuration AA-2 is the most energetically favorable one with the minimum binding energy. The change of the binding energy with respect to the layer spacing between SiC and AlN monolayers is depicted in Fig. 1g. The layer spacing for which the binding energy is minimum is the optimum layer spacing (D). Table S1 enrolls the values of optimized interlayer spacing for six patterns. Stacking configuration AA-2 has the minimum inter-layer spacing between the SiC and AlN layer with the greatest negative binding energy (i.e., the most stable configuration). However, there is no covalent bond between the SiC monolayer and the AlN monolayer. The absence of covalent bond is confirmed by calculating the bond length between the atoms of the two different layers. Taking the AA-2 configuration with the minimum optimum layer spacing (2.31 Å), the sum of the covalent radius of Si and N is 1.87 Å (1.16 + 0.71 = 1.87) while the sum is 2.01 Å for Al and C atom (1.26 + 0.75 = 2.01)^74^. The summed values are less than 2.31 Å (for other configurations the equilibrium layer spacing is more than 3 Å), which eliminates the prospect of creating any covalent bond between the SiC and AlN layer. Besides, the binding energy per interface area renders quantitative information on the type of interaction between the two layers of the heterostructure. Taking the most stable AA-2 configuration, the binding energy per interface area is −69.9 meV/Å^2^, which suggests that weak van der Waals forces are predominating in the heterostructure^75,76^. This result is comparable to the ZnO/GaN vdW heterostructure studied by Ren et al*.*^73^. For the most energetically favorable AA pattern of the ZnO/GaN vdW heterostructure, they reported −60.77 meV/Å^2^ binding energy and 2.41 Å interface distance ^73^ along with weak vdW interactions in the heterostructure^73^. Nguyen et al*.*^77^ also reported similar binding energy for Boron Phosphide/MoGe_2_N_4_ vdW heterostructure (−67.28 meV/Å^2^ for their most stable ‘stacking-II’ pattern) but with a higher interlayer spacing 3.08 Å. In this context, He et al*.*^78^ reported an interlayer distance of 2.604 Å between InSe and g-C_3_N_4_ monolayers for the most energetically favorable H_N1_ configuration while investigating InSe/g-C_3_N_4_ heterostructure, comparable to our AA-2 configuration.

**Figure 2:**
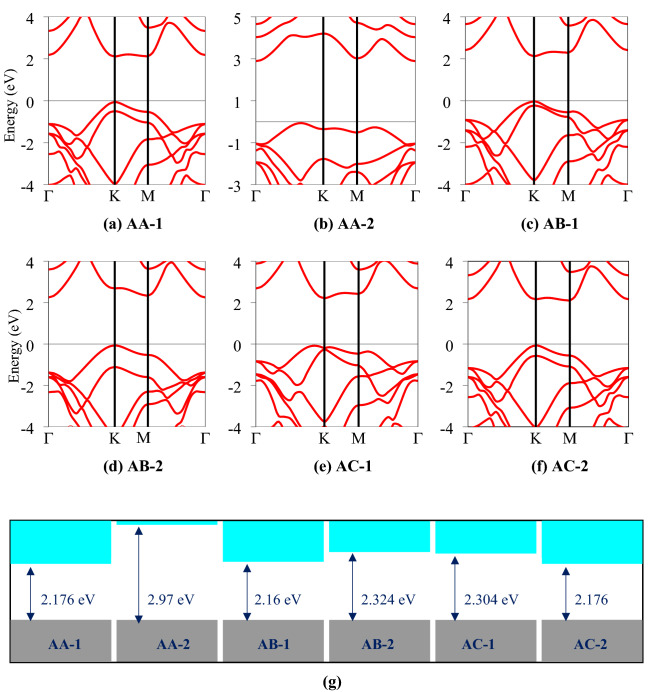
Electronic band diagrams of six stacking configurations of the SiC/AlN van der Waals heterostructure: (**a**) AA-1, (**b**) AA-2, (**c**) AB-1, (**d**) AB-2, (**e**) AC-1, (**f**) AC-2. (**g**) Calculated electronic band gaps of the six configurations of the heterostructure.

**Figure 3:**
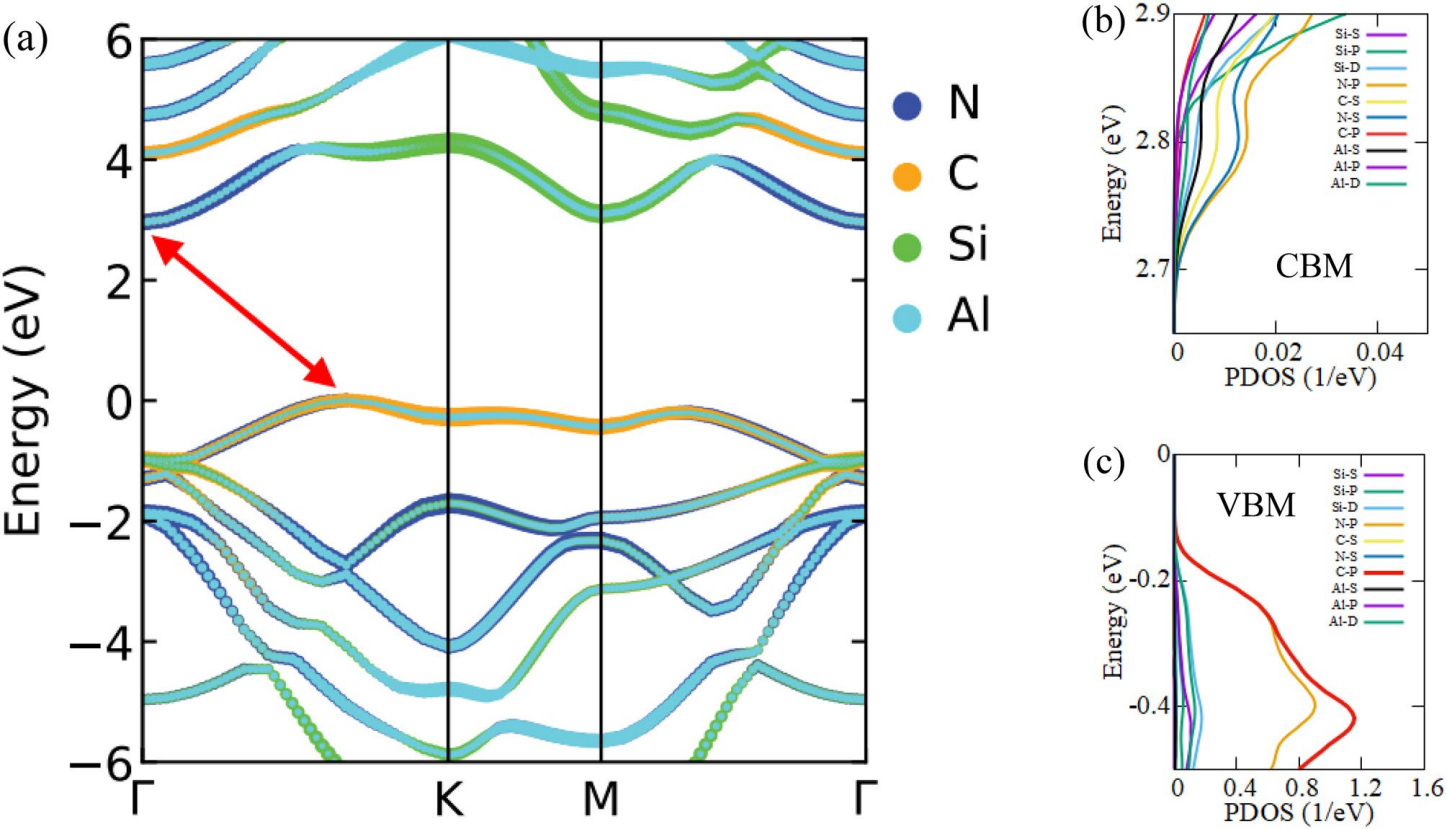
(**a**) Projected band structure of the SiC/AlN bilayer heterostructure for the most stable AA-2 configuration with the contributions from the N, C, Si, and Al atoms are represented by navy blue, cyber yellow, green and sky-blue colors, respectively. Magnified view of the partial density of states (PDOS) around the (**b**) CBM, and the (**c**) VBM.

The electronic band structures of the six configurations of the SiC/AlN vdWBH are portrayed in Fig. 2, while the electronic band gaps and the relevant VBM and CBM positions of the six structures are enlisted in Table S1. The AA-2 pattern (most energetically favorable configuration) demonstrates an indirect bandgap of 2.97 eV. The VBM is at the $\Gamma$–K route while the CBM is placed at the high symmetry $\Gamma$ point. For our subsequent calculations, among the six stacking configurations of the SiC/AlN vdWBH, we concentrated on the AA-2 configuration i.e., the most stable stacking configuration. In order to study the band alignment of the SiC/AlN vdWBH, the projected band structure and the density of states (DOS) of the heterostructure (AA-2 configuration) are examined. The projected band structure of the heterostructure along with the contributions from the Si, C, Al, and N atoms is shown in Fig. 3a. Obviously, the C atom dominates in the VBM of the heterostructure (indicated by the cyber yellow color in the projected band diagram), while the CBM is provided mostly by the N atom (represented by the navy-blue color in the figure), which confirms the formation of the typical type-II band orientation in the SiC/AlN heterostructure. To analyze the nature of the electronic states at VBM and CBM to a greater extent, the partial density of states (PDOS) of the SiC/AlN vdWBH is calculated. Enlarged views of the PDOS around the CBM and VBM are shown in Fig. 3b,c, respectively. Clearly, the CBM is mostly made up of the N-p and N-s states, while the VBM is dominated by the C-p state. This further confirms that the SiC/AlN heterostructure has a typical type-II band alignment. Photogenic electrons and holes will accumulate on the surfaces of the AlN sheet and SiC sheet, respectively, leading to indirect excitons. Consequently, the type-II band configuration of the SiC/AlN vdWBH separates the photogenic electrons and holes effectively in real space. The heterostructure, therefore, appears to be a promising candidate for solar-energy-driven water decomposition as well as high-performance optoelectronic devices which prevent electron–hole recombination^37^.

The work function ($\Phi$) is an important quantity generally utilized as an inherent reference for band alignment^79,80^. It is the least energy necessary to transfer an electron from the Fermi energy level to the free space. The following relation is utilized to calculate the work function of a semiconductor:

5 $$ \Phi= {\text{E}}_{\text{vacuum}}-{\text{E}}_{\text{Femi}}$$

where E_Fermi_ being the Fermi energy and E_vacuum_ refers to a static electron’s energy in the free space close to the surface. Fermi energy is determined from the calculation of the ground-state electronic structure. One can estimate the value of E_vacuum_ from the plot of the average electrostatic potential for a surface calculation employing an adequate amount of vacuum in the unit cell. Fig. S2a–c, respectively illustrates the average electrostatic potential plot of the SiC monolayer, AlN sheet and SiC/AlN vdWBH. From expression (5), we obtained the work functions values as 5.06 eV, 5.38 eV and 5.31 eV for 2D SiC, AlN monolayer and SiC/AlN vdWBH, respectively. When the SiC sheet and the AlN layer are in close contact, because of the Fermi level of the SiC being higher than that of AlN, electrons will spontaneously diffuse from the SiC monolayer to the AlN monolayer. The Fermi level of SiC will shift downward gradually while the Fermi level of AlN will shift upward in accord with the rise in the number of transported electrons and ultimately achieves the same level in the system, which is responsible for the 5.31 eV work function of the heterostructure. Figure S2(c) also suggests similar characteristics which is attributable to the higher potential energy of the SiC layer in contrast to the AlN layer. Therefore, positive charges will aggregate on the SiC sheet while the negative charges will aggregate on the AlN sheet and consequently a built-in electric field (E_i_) will be formed, directed from the SiC layer to the AlN layer. The electric field, E_i_ stimulates the inside carrier drift of the SiC/AlN vdWH and eventually an equilibrium is achieved through the diffusion force.

Suitable band edge positions of the semiconducting material are crucial to induce photocatalytic water disassociation reaction. We, therefore, explore the band edge locations of the SiC/AlN vdWBH to examine its potential in the solar-to-hydrogen conversion process. The band alignments of the semiconducting materials are calculated using the relations^26,36,81^:

6 $${\text{E}}_{\text{VBM}}{=}-\text{X}-\frac{\text{Eg}}{{2}}$$

7 $${\text{E}}_{\text{CBM}}{=}-\text{X}+\frac{\text{Eg}}{{2}}$$

where E_VBM_ and E_CBM_ refer to the VBM and CBM energy levels of the semiconductor, respectively. ${\rm X}$ denotes the material’s Mulliken electronegativity, which can be determined by taking the geometric mean of the Mulliken electronegativities of the component atoms^82,83^. ${\text{Eg}}$ is the electronic bandgap of the corresponding material. The Mulliken electronegativities for Si, C, Al and N atom are 4.76, 6.27, 3.21 and 7.27, correspondingly^84^. Our calculation yielded the values of ${\rm X}$ as 5.46, 4.83 and 5.14 for the isolated SiC layer, AlN sheet, and SiC/AlN vdWBH, respectively. Generally, a semiconductor must meet certain requirements in order to generate hydrogen (H_2_) and oxygen (O_2_) through photocatalysis: (a) the energy level of the VBM of the semiconductor must be equal to or below −5.67 eV from the vacuum level (oxidation potential) to generate O_2_ through oxidation reaction (O_2_/H_2_O) at pH  0 environment. (b) CBM energy level should be at least −4.44 eV from the vacuum (reduction potential) to generate H_2_ through the reduction reaction (H^+^/H_2_) at pH  0. (c) An electronic bandgap of a minimum of 1.23 eV of the semiconductor material. (d) The material should possess notable absorption peaks in the ultra-violet (UV) and visible region of the solar energy spectrum to use much of the solar energy and (e) A high ratio of the surface to the volume of the material to promote the photocatalytic action.

**Figure 4:**
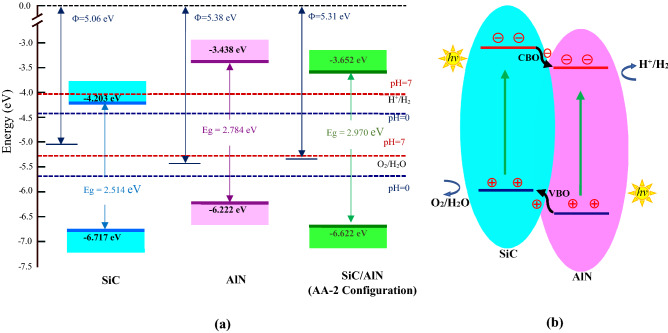
(**a**) Band edge locations of free-standing the SiC layer, the AlN monolayer, and the SiC/AlN heterostructure. (**b**) Schematic illustration of the photocatalytic action of the SiC/AlN van der Waals heterostructure with the transfer of the photogenic carriers at the interface. VBO and CBO refer to the valence band offset and conduction band offset, respectively.

The alignments of the energy levels for isolated SiC sheet, free standing single layered AlN and SiC/AlN vdWBH are demonstrated in Fig. 4a. It is important to note that the pH value of the environment inevitably affects the potential of oxidation and reduction reactions. One can determine the standard reduction potential of the hydrogen evolution reaction (H^+^/H_2_) by the formula E_red_ = −4.44 eV + pH $\times$ 0.059 eV while the standared oxidation potential can be obtained using the relation E_ox_ = −5.67 eV + pH $\times$ 0.059 eV. The redox potentials for a neutral environment (pH  7) were also analyzed, indicated by red dotted lines in Fig. 4a. As the CBM and VBM of the SiC sheet and AlN layer suggest in Fig. 4a, the energy gap of 2D-SiC extends from −6.717 to −4.203 eV while for 2D AlN the energy gap extends from −6.222 to −3.438 eV. The AlN monolayer suits photocatalytic water splitting action for both pH  0 (acidic) and pH  7 (neutral) environments, but the SiC sheet does not satisfy the condition of water splitting at neutral environment. Nevertheless, the bandgap of the SiC/AlN vdWBH spans from −6.622 to −3.652 eV which satisfies the condition for photocatalytic water disassociation in both the neutral and the acidic environment. The free-standing SiC layer and AlN layer do not have the type-II band configuration to limit the photogenic carrier recombination and consequently, the SiC/AlN vdWBH can be a promising candidate for photo-catalysis separating the photogenic electrons and holes and inducing the water decomposition reactions to produce oxygen and hydrogen.

Figure 4b describes the mechanism of solar energy driven water decomposition in the SiC/AlN vdWH along with the migration of photo-generated carriers in the type-II band orientation. When the heterostructure will be illuminated by the solar irradiation and photon energy will be higher than the bandgap value of SiC and AlN, excited electrons will start migrating from the valence band to the conduction band of SiC and AlN sheets, leaving behind holes in the valence band. Once the photo-generated electrons reach the conduction band of SiC, due to the promotion of conduction band offset (CBO), they will move to the conduction band of AlN. On the other hand, owing to the promotion of the valence band offset (VBO), holes created in the valence band will start moving from AlN to SiC simultaneously. The CBO has been calculated as 0.28 eV, while the VBO is 0.55 eV. Accordingly, photo-excited positive and negative charge carriers are effectively separated across the heterojunction and will take part in the photocatalytic redox reactions. Hydrogen will be produced in the AlN sheet while oxygen production will take place at the SiC layer.

**Figure 5:**
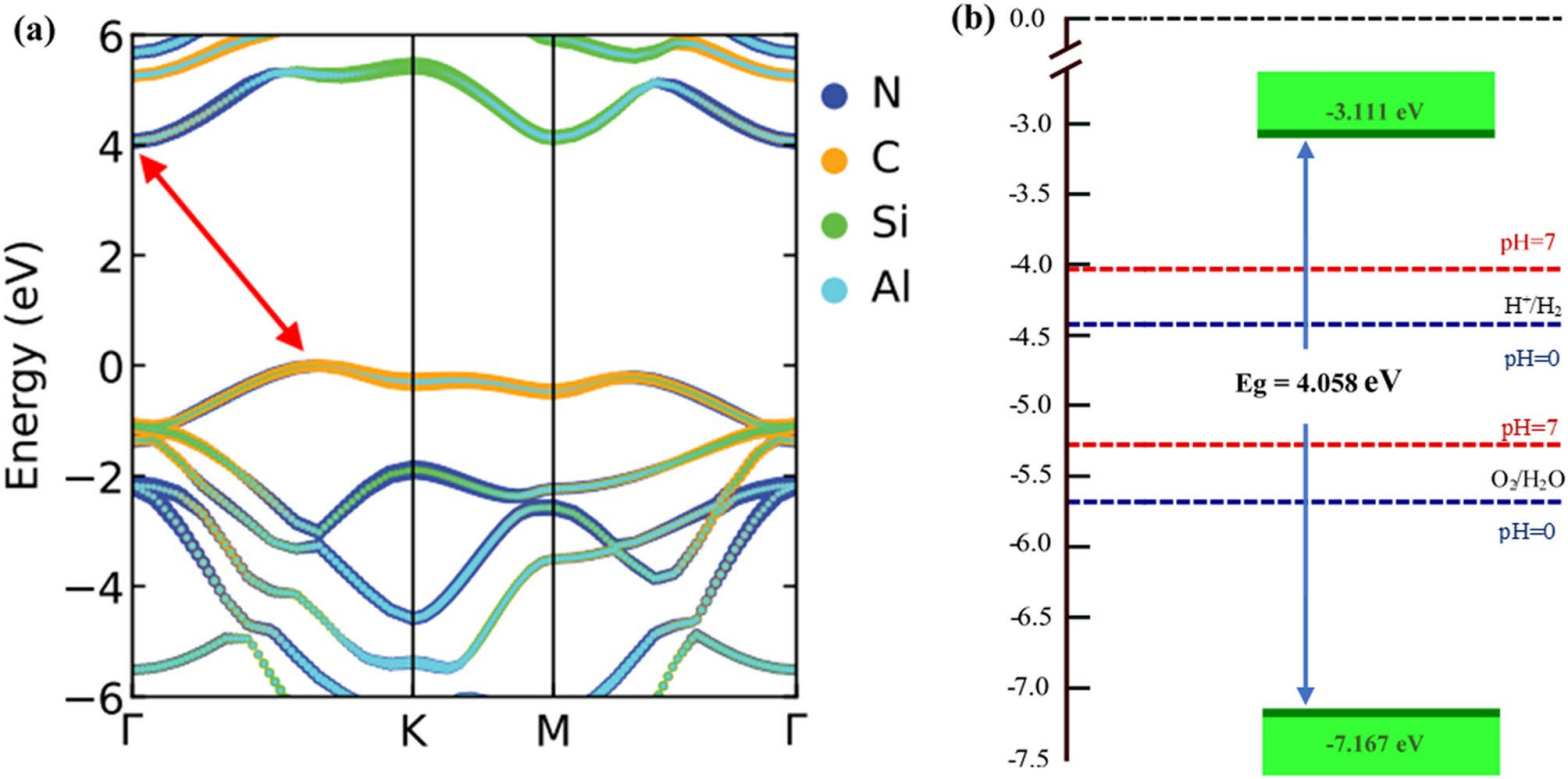
(**a**) Projected band structure of SiC/AlN van der Waals heterostructure (AA-2 configuration) calculated using HSE06 hybrid functional. (**b**) Relevant band edge positions of the heterostructure obtained after HSE06 band structure calculation.

As HSE06 functional is a well-known hybrid function that yields an electronic bang-gap value close to the experiment, we have also employed HSE06 functional for a more exact electronic structure calculation^85^. HSE06 calculated projected band diagram for the AA-2 configuration of the SiC/AlN vdWH is illustrated in Fig. 5a. We obtained a 4.058 eV electronic band gap for the AA-2 structure employing the HSE06 functional. The contributions from four different atoms are represented by four different colors. It is apparent that VBM comes mostly from the C atom (represented by the cyber yellow color), while the N atom dominates in the CBM (indicated by the cyber yellow color). Thus, the formation of type-II band alignment for SiC/AlN vdWBH is again confirmed from the HSE06 calculated band diagram. In addition, the relevant band edge positions are also calculated and depicted in Fig. 5b. The band gap extends from −7.169 to −3.111 eV for the HSE06 functional, ensuring solar energy-driven water splitting for both pH  0 and pH  7 environments.

**Figure 6:**
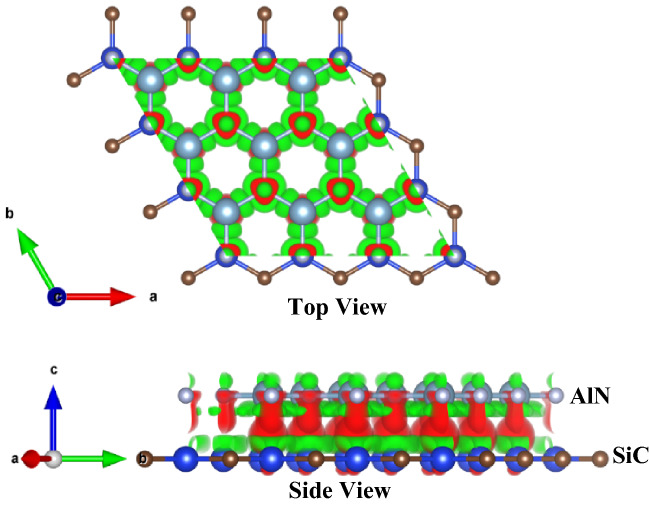
A 3D plot of the charge density difference of the SiC/AlN heterobilayer where the red and the green indicate the areas where the charges are accumulated and depleted, respectively. The isovalue is 0.00286 e/Å^3^.

Furthermore, in order to characterize the charge transfer process and charge distribution between the SiC layer and the AlN layer after the formation of the SiC/AlN vdWBH, the charge density difference (CDD) of the heterobilayer is estimated as:

8 $$ \Delta \rho = \rho_{\text{SiC/AlN}}-{\rho}_{\text{SiC}}-{\rho}_{\text{AlN}}$$

where $\Delta \rho$ is the CDD of the heteostructure, ${\rho}_{\text{SiC/AlN}}$, ${\rho}_{\text{AlN}}$ and ${\rho}_{\text{SiC}}$ stand for the charge density of the SiC/AlN vdWBH, free standing AlN layer, and isolated SiC layer, respectively. Figure 6 shows the CDD of the SiC/AlN vdWBH. The red color in the CDD plot represents the area where charge is accumulated, and the green color is for the region where charge is depleted. As the CDD plot suggests, electron has been accumulated at the interface area, mainly near the AlN layer, while it has been depleted mostly from the SiC layer in the SiC/AlN heterostructure. This characteristic indicates that charge will be transmitted from the SiC sheet to the AlN sheet. Charge redistribution at the interface area generates an inherent electric field directed from the SiC layer to the AlN layer. This inherent electric field plays an essential role in separating the photogenic electrons and holes and prolonging their lifetime^86,87^. This feature is in line with the findings of the average electrostatic potential in Fig. S2c.

**Figure 7:**
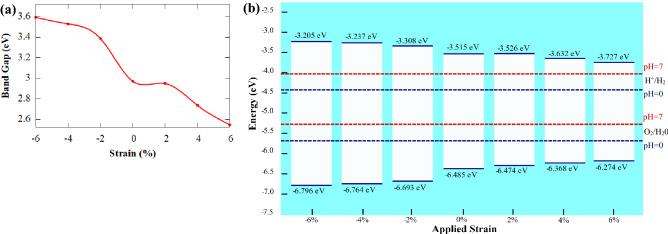
(**a**) Variation of the bandgap energy of the SiC/AlN heterobilayer (AA-2 Configuration) with respect to the external biaxial strain applied to the structure. (**b**) Evolution of the band edge position of the heterostructure for the AA-2 configuration as a function of the varying percentages of the biaxial strain.

Strain engineering is regarded as an efficient approach for modifying the electrical, optical, as well as transport behavior of the semiconductors^88^. In particular, one-dimensional, and two-dimensional crystal forms can withstand much higher strain than their bulk analogues. Hence, the in-plane biaxial strain is incorporated into the SiC/AlN vdWBH to investigate how the in-plane strain influences the band configurations and the electronic band gaps of the heterostructure system. The strain has been applied to the heterostructure using the relation:

9 $$e=\frac{{a}_{strained}-{a}_{relaxed}}{{a}_{relaxed}}\times 100\%$$

where ${a}_{strained}$ and ${a}_{relaxed}$ refer to the lattice constant of the heterostructure under biaxial strain and the equilibrium lattice constant, respectively while *e* denotes the percentage of strain applied. The strain has been applied bi-axially to the heterostructure in the range −6% to the + 6% with a step of 2%. The strained band structures of different percentages of strain are depicted in Fig. S3. Figure 7a illustrates the variation of the electronic band gap with respect to the percentage strain incorporated. Clearly, tensile strain decreases the bandgap while compressive strain increases it. Therefore, when the heterostructure is subject to tensile strain, owing to the reduction in the electronic band-gap, the heterostructure absorption profile enhances from the UV to visible spectrum and promotes photocatalytic performance. The energetic positions of the VBM and CBM when the SiC/AlN vdWBH is under biaxial strain are shown in Fig. 7b. As Fig. 7b suggests, the heterostructure ensures a favorable band alignment for the redox reaction in both neutral (pH  7) and acidic (pH  0) environments under strain in the range −6% to 6%. The location of the VBM increases while the CBM location decreases linearly upon applying the strain from −6% to the + 6%. However, the band edges are in a proper energetic position to initialize the photocatalytic redox reaction for water decomposition. This type of desired band edge alignments of the SiC/AlN heterostructure indicates strong strain tolerance of solar-energy-driven water decomposition ability.

**Figure 8:**
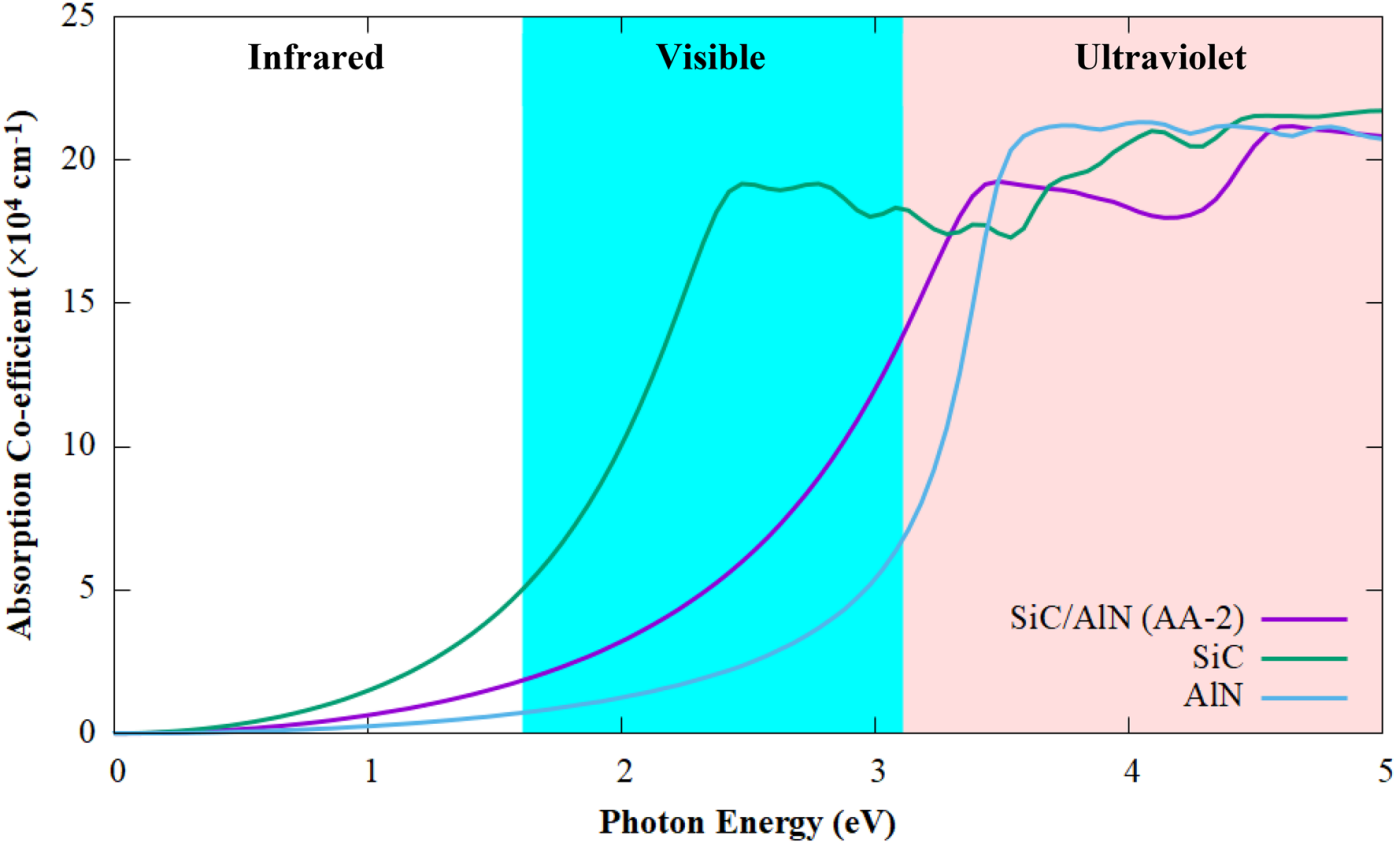
Optical absorption spectra of the SiC/AlN van der Waals heterostructure (AA-2 configuration) along with that of the free-standing SiC monolayer and the AlN monolayer.

The absorption spectrum of the material provides insight into the optical properties of the material. Figure 8 shows the absorption profile of the SiC/AlN vdWBH. The absorption coefficient of its constituents, SiC monolayer and AlN monolayer is also included for comparison. Clearly, the SiC/AlN heterostructure shows a broad optical absorption spectrum, spanning from the UV to the near-infrared (IR) regime. In particular, the heterostructure shows a high UV absorption coefficient, reaching up to 2.16 $\times$ 10^5^ cm^−1^. Furthermore, compared to the AlN monolayer, the heterostructure exhibits a substantial enhancement in optical absorption in the visible light zone. The SiC/AlN heterostructure is therefore an excellent solar irradiation absorber facilitating highly efficient photo-catalysis activity. Higher intensity and increased number of absorption points in the structure result in a higher photo-catalysis reaction and increased production of electron–hole pairs. In contrast, applying strain to the structure biaxially can cause its UV absorption profile to shift towards the visible region, allowing for a more effective deployment of the solar spectrum. We, therefore, calculated the absorption coefficient of the SiC/AlN vdWBH when the heterostructure is subject to biaxial strain. The absorption profile of the heterostructure under varying strain from −6 to + 6% is depicted in Fig. S4. Obviously, tensile strain improves the visible light absorption profile by increasing the absorption coefficient in this region. At + 6% strain, the visual absorbance is significantly enhanced compared to the unstrained structure. This is consistent with the electronic band gap values when the heterostructure is under tensile strain. In contrast, compressive strain shifts the absorption spectrum towards the UV range with the reduction in the magnitude of absorption coefficient in the visible regime. Thus, the optical performance of the SiC/AlN vdWBH can be mediated effectively through strain engineering with tensile strain improving the visible spectrum absorption of the heterojunction.

## Conclusion

The photocatalytic potential of the SiC/AlN bilayer heterostructure has been studied thoroughly in the water decomposition process by employing first-principles calculations. The SiC/AlN vdWBH possesses an inherent type-II band configuration where the VBM (CBM) is contributed by the SiC sheet (AlN sheet), promoting efficient spatial separation of the photogenic carriers. Investigation of the band alignments reveals that the heterostructure has adequately large kinetic overpotentials to provoke the redox reaction to disassociate the water into its constituents under neutral as well as acidic environments. Due to the variation in work-function values, band edge positions are altered substantially with the formation of the heterostructure. Photoexcited electrons (holes) accumulate on the AlN (SiC) layer. The oxygen production reaction and the hydrogen production reaction take place separately at the SiC layer the AlN layer, respectively. The electric field at the interface formed due to the significant amount of charge transport prevents photogenic electron–hole recombination, further improving photocatalytic performance. Ample optical absorption of the SiC/AlN heterostructure extending from the UV to the near-infrared regime indicates the strong potential of the heterostructure in solar energy harvesting water disassociation. Interestingly, strain can mediate the optical characteristics of the vdWH, with an increase in tensile strain resulting in the enhancement of the absorption profile in the visible regime. The collective outcome of these intriguing features highlights the enormous potential of the SiC/AlN vdW heterostructure in 2D excitonic photo-catalysis.

## Supplementary Information

Supplementary Information.

## Data Availability

The datasets used and/or analyzed during the current study available from the corresponding author on reasonable request.
