# Supplementary material for: Two-dimensional SiC/AlN based type-II van der Waals heterobilayer as a promising photocatalyst for overall water disassociation
Source: Sci Rep. 2022 Nov 22;12:20106. doi: 10.1038/s41598-022-24663-y (PMC9684528; doi:10.1038/s41598-022-24663-y)
Supplement: Supplementary file 1 — Supplementary Information. [file 41598_2022_24663_MOESM1_ESM.docx]

**Supplementary Materials**

**Two-dimensional SiC/AlN based type-II van der Waals heterobilayer as a promising photocatalyst for overall water disassociation**

Naim Ferdous^1^, Md. Sherajul Islam^1,2^*, Jeshurun Biney^1^, Catherine Stampfl^3^ & Jeongwon Park^1,4^

^1^Department of Electrical & Biomedical Engineering, University of Nevada, Reno, NV 89557, USA.

^2^Department of Electrical and Electronic Engineering, Khulna University of Engineering & Technology, Khulna 9203, Bangladesh.

^3^School of Physics, The University of Sydney, Sydney, NSW 2006, Australia.

^4^School of Electrical Engineering and Computer Science, University of Ottawa, Ottawa, ON K1N6N5, Canada.

*Corresponding author. Email: [sheraj_kuet@eee.kuet.ac.bd](mailto:sheraj_kuet@eee.kuet.ac.bd)

**Table S1:** Calculated binding energy (meV/$Å^{2})$, optimized interlayer distance ($Å)$, electronic band-gap calculated using optB86b-vdW (eV) and the positions of the valence band maximum (VBM) and the conduction band minimum (CBM) for the six configurations of the SiC/AlN bilayer heterostructure.

| Stacking Configuration | Binding energy (meV/$Å^{2})$ | Optimized interlayer distance ($Å)$ | Band gap (calculated with optB86b-vdW in eV) | VBM Position | CBM Position |
| --- | --- | --- | --- | --- | --- |
| AA-1 | -15.11 | 3.98 | 2.176 | K point | K point |
| AA-2 | 69.9 | 2.31 | 2.97 | $\Gamma$-K route | $\Gamma$ point |
| AB-1 | 20.7 | 3.57 | 2.16 | K point | K point |
| AB-2 | 29.97 | 3.11 | 2.324 | K point | $\Gamma$ point |
| AC-1 | 32.38 | 3.07 | 2.304 | $\Gamma$-K route | K point |
| AC-2 | -16.13 | 3.89 | 2.176 | K point | M point |


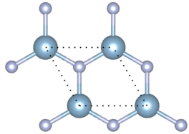

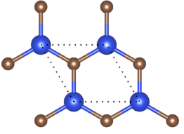


1. **(b)**


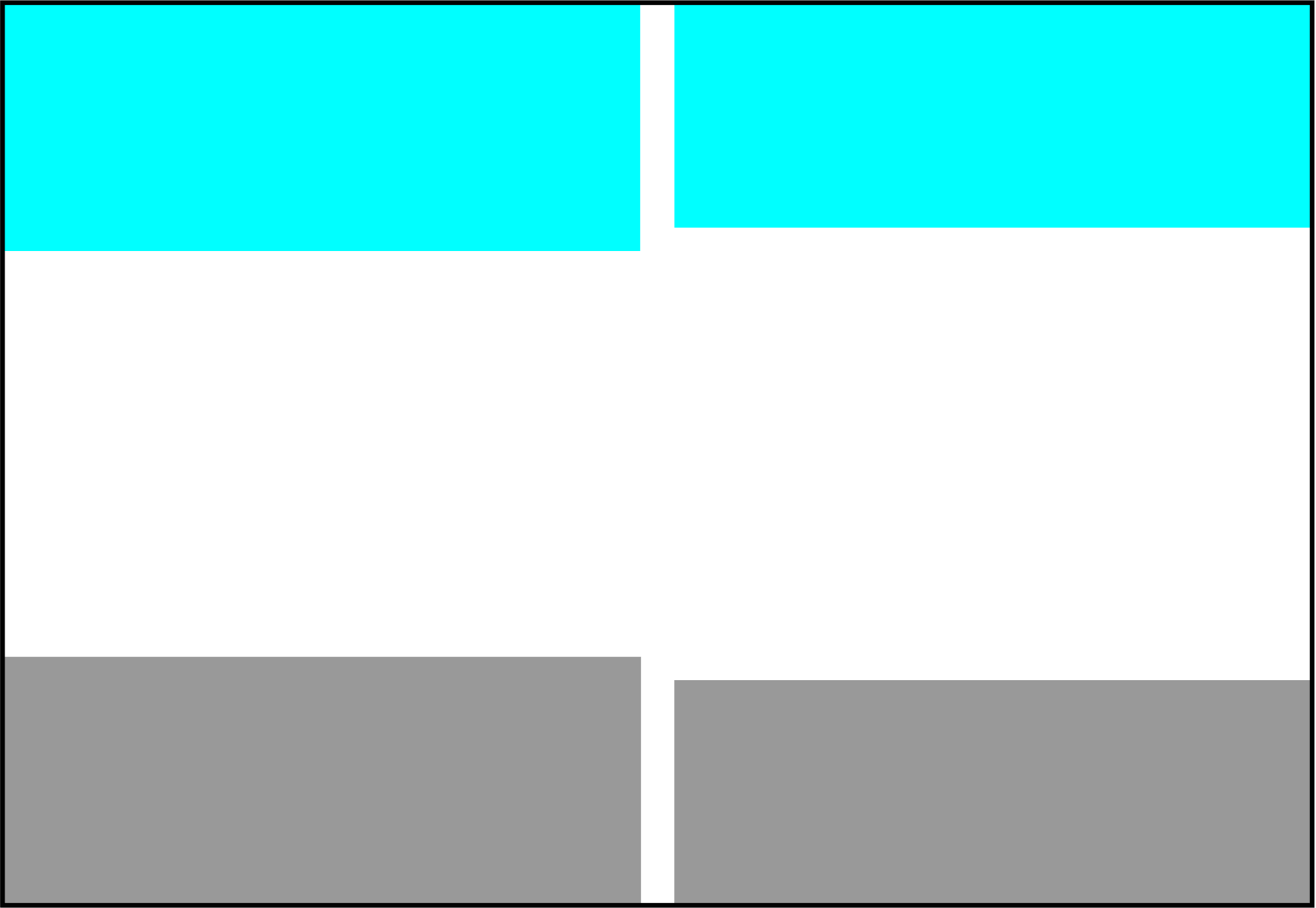


2.514 eV 2.7844 eV

**2D SiC 2D AlN**

**(c)**

**Figure S1.** Calculated electronic band structures of (a) the free-standing SiC monolayer, and (b) the isolated AlN layer. The insets show the top views of the SiC monolayer and the AlN monolayer. (c) Obtained electronic band gaps for the pristine SiC sheet and AlN sheet.


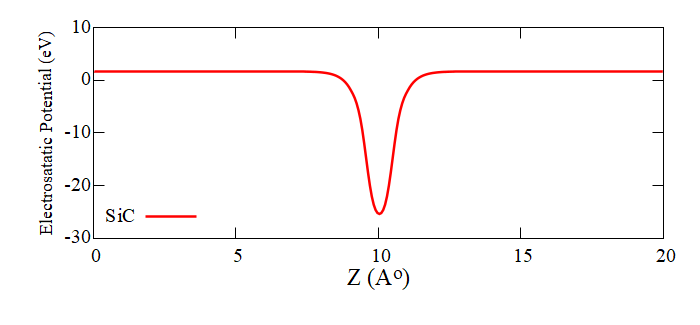

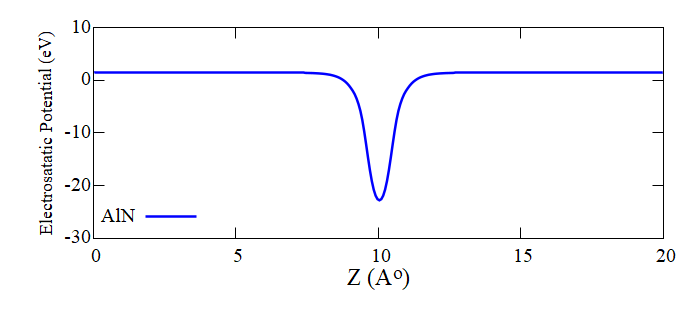

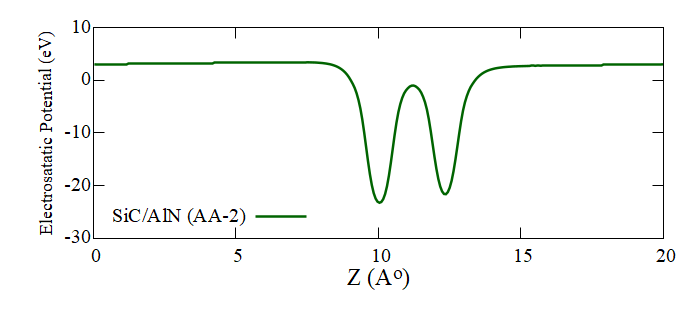


**(a)**

**(b)**

**(c)**

SiC

AlN

**E_i_**

**Figure S2.** The plane average electrostatic potential of (a) the SiC monolayer, (b) the AlN layer, and (c) the SiC/AlN van der Waals heterostructure. E_i_ refers to the built-in electric field at the heterostructure interface pointing from the SiC layer to the AlN layer.

Energy (eV)

1. **+2% Strain (b) +4% Strain (c) +6% Strain**

Energy (eV)

**(d) -2% Strain (e) -4% Strain (f) -6% Strain**

**Figure S3.** Band structures of the SiC/AlN van der Waals heterostructure for the AA-2 configuration under varying biaxial strain: (a) +2% strain (b) +4% strain (c) +6% strain (d) -2% strain (e) -4% strain (f) -6% strain.


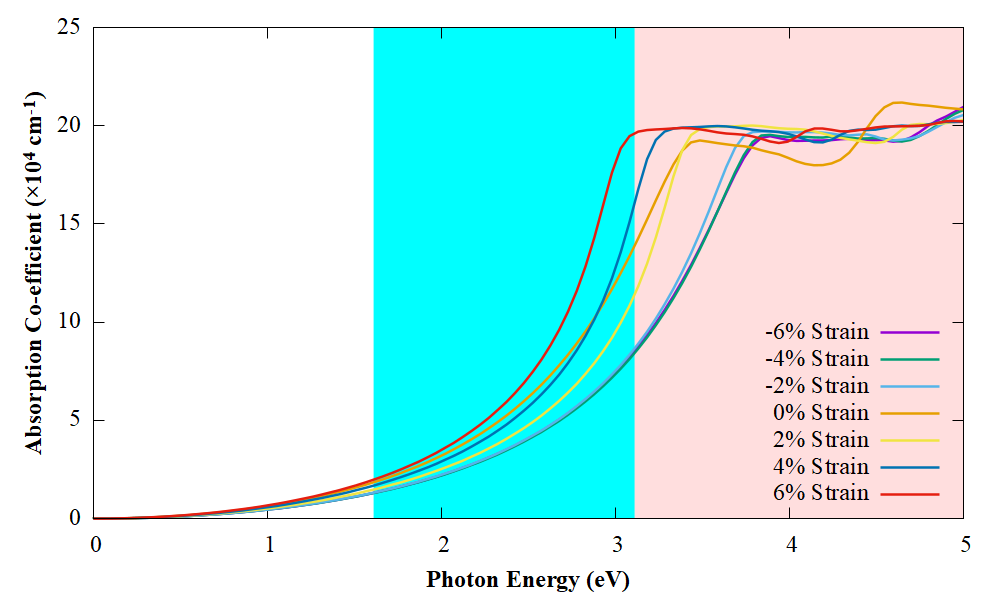


**Infrared Visible Ultraviolet**

**Figure S4.** Optical absorption spectra of the heterostructure (AA-2 configuration) with various percentages of biaxial strain applied to the heterostructure.
